# Disruption of Glycerol Metabolism by RNAi Targeting of Genes Encoding Glycerol Kinase Results in a Range of Phenotype Severity in *Drosophila*

**DOI:** 10.1371/journal.pone.0071664

**Published:** 2013-09-06

**Authors:** Patrick J. Wightman, George R. Jackson, Katrina M. Dipple

**Affiliations:** 1 Department of Human Genetics, David Geffen School of Medicine at University of California Los Angeles, Los Angeles, California, United States of America; 2 Department of Neurology, David Geffen School of Medicine at University of California Los Angeles, Los Angeles, California, United States of America; 3 Brain Research Institute, Semel Institute for Neuroscience and Human Behavior, David Geffen School of Medicine at University of California Los Angeles, Los Angeles, California, United States of America; 4 Center for Neurobehavioral Genetics, Semel Institute for Neuroscience and Human Behavior, David Geffen School of Medicine at University of California Los Angeles, Los Angeles, California, United States of America; 5 Department of Pediatrics, David Geffen School of Medicine at University of California Los Angeles, Mattel Children's Hospital at University of California, Los Angeles, Los Angeles California, United States of America; Thomas Jefferson University, United States of America

## Abstract

In *Drosophila*, RNAi targeting of either *dGyk* or *dGK* can result in two alternative phenotypes: adult glycerol hypersensitivity or larval lethality. Here we compare these two phenotypes at the level of glycerol kinase (GK) phosphorylation activity, *dGyk* and *dGK*-RNA expression, and glycerol levels. We found both phenotypes exhibit reduced but similar levels of GK phosphorylation activity. Reduced RNA expression levels of *dGyk* and *dGK* corresponded with RNAi progeny that developed into glycerol hypersensitive adult flies. However, quantification of *dGyk*/*dGK* expression levels for the larval lethality phenotype revealed unexpected levels possibly due to a compensatory mechanism between *dGyk* and *dGK* or RNAi inhibition. The enzymatic role of glycerol kinase converts glycerol to glycerol 3-phosphate. As expected, elevated glycerol levels were observed in larvae that went on to develop into glycerol hypersensitive adults. Interestingly, larvae that died before eclosion revealed extremely low glycerol levels. Further characterization identified a wing phenotype that is enhanced by a *dGpdh* null mutation, indicating disrupted glycerol metabolism underlies the wing phenotype. In humans, glycerol kinase deficiency (GKD) exhibits a wide range of phenotypic variation with no obvious genotype-phenotype correlations. Additionally, disease severity often does not correlate with GK phosphorylation activity. It is intriguing that both human GKD patients and our GKD *Drosophila* model show a range of phenotype severity. Additionally, the lack of correlation between GK phosphorylation and *dGyk*/*dGK*-RNA expression with phenotypic severity suggests further study including understanding the alternative functions of the GK protein, could provide insights into the complex pathogenic mechanism observed in human GKD patients.

## Introduction

Glycerol kinase (GK) is an enzyme that catalyzes the conversion of glycerol to glycerol 3-phosphate in an ATP dependent reaction [1]. It plays an important role in both human metabolism and development as shown by the symptoms of glycerol kinase deficiency (GKD [MIM 307030]). Patients with GKD can have isolated hyperglyceroluria and hyperglyceremia, or severe CNS and metabolic abnormalities [2], [3]. Patient studies have revealed an absence of genotype-phenotype correlations [2], [4], [5]. Additionally, the severity of GKD patient symptoms does not always correlate with GK phosphorylation activity [2]. This suggests the existence of a complex pathogenic mechanism that could involve a role for genetic modifier loci [2], [6]–[9] or alternative functions of the GK protein [10], [11] such as the ATP stimulated translocation of the glucocorticoid receptor [12], [13]. Although the mouse model for GKD displays neonatal death [14], [15], study of this mouse model has revealed a role for glycerol kinase in apoptosis [16] in addition to altered expression of gene networks involved in lipid metabolism, carbohydrate metabolism, and insulin signaling [17], [18]. Here, we evaluate the potential of a *Drosophila* GKD model [19] by looking for molecular or metabolic similarities with GKD in humans.

RNAi targeting of the *Drosophila* glycerol kinase genes *dGyk* (CG18374) or *dGK* (CG7995) results in two alternative phenotypes: larval lethality or glycerol hypersensitive adult flies [19]. Previously, the analysis of 3^rd^ instar larvae that developed into glycerol hypersensitive adults revealed successful targeting of *dGyk* and *dGK* that correlated with reduced glycerol kinase phosphorylation activity and elevated glycerol levels. Glycerol hypersensitive flies die rapidly when placed on a food source supplemented with glycerol, and sensitivity is enhanced by null mutations in eye pigmentation genes [19]. The glycerol hypersensitive phenotype suggests the flies are unable to tolerate the strong hydrophilic properties of glycerol in the food media. Insects are highly sensitive to desiccation [20], [21], and *in vivo* glycerol has been shown to play an important role in the control of water balance and insect desiccation resistance [22].

We hypothesized that phenotypic severity would correlate with glycerol kinase phosphorylation activity and expression level of the RNAi target gene. Therefore, we compared glycerol kinase phosphorylation, *dGyk*- and *dGK*-RNA expression and glycerol levels in 3^rd^ instar larvae for both glycerol hypersensitive and larval lethality phenotypes. This analysis revealed GK phosphorylation levels were reduced but similar for both phenotypes. Further analysis detected distinct *dGyk* and *dGK* expression patterns between the two phenotypes. As expected, elevated glycerol levels were detected in 3^rd^ instar larvae that went on to develop into glycerol hypersensitive flies. However, 3^rd^ instar larvae that died before eclosion had below normal levels of glycerol, suggesting the existence of a deleterious metabolic pathway. Additionally, a crumpled wing phenotype was produced by RNAi targeting of *dGyk*, the severity of which was enhanced by a null mutation of the glycerol 3-phosphate dehydrogenase (*dGpdh*) gene, the next step in the glycerol metabolism pathway, indicating that this wing phenotype was caused by disrupted glycerol metabolism.

We propose that the lack of correlation between RNAi phenotype severity with glycerol kinase phosphorylation activity, *dGyk*- and *dGK*-RNA expression levels, and glycerol levels is similar to the complexity observed in GKD clinical studies. Therefore further study of this *Drosophila* model for GKD could provide powerful insight into the complex pathogenic mechanism that underlies the wide range of phenotype severity observed in human GKD patients.

## Results

### RNAi targeting of *dGyk* or *dGK* can result in larval lethality or glycerol hypersensitive adult flies

Analysis of RNAi fly lines targeting *dGyk* or *dGK* expression, named *dGyk*-IR and *dGK*-IR respectively (IR: inverse repeat) was initially performed using a *Tubulin*-GAL4 (*Tub*-GAL4) driver for ubiquitous expression of the inserted construct. Each RNAi fly line (9 and 10 each for *dGyk*-IR and 10× *dGK*-IR, respectively) was crossed to the *Tub*-GAL4 driver fly line and the progeny examined for physical phenotypes [19]. Progeny from these crosses could be divided into two groups: survival to adulthood with no obvious physical phenotype (named *dGyk*-IR-sur and *dGK*-IR-sur) or lethality during larval development (named *dGyk*-IR-let and *dGK*-IR-let). Adult flies were subsequently found to be hypersensitive to glycerol [19]. Results were confirmed in at least 2 fly lines for each phenotype and in alternative gene regions for RNAi targeting [19]. Initial phenotypic characterization is summarized in Table S1.

We have previously shown that GFP levels (the *pUds*GFP RNAi vector co-expresses GFP) are greater in *dGyk*-IR-let/*Tub*-GAL4 3^rd^ instar larvae as compared to *dGyk*-IR-sur; *Tub*-GAL4 3^rd^ instar larvae [19]. A similar trend was observed for *dGK*-IR-let/*Tub*-GAL4 compared to *dGK*-IR-sur; *Tub*-GAL4 3^rd^ instar larvae. This indirect measure of the inverse repeat (IR) expression levels suggested that the larval lethality phenotype was due to greater expression of the IR expression construct and consequently lower levels of *dGyk* or *dGK*. Here we characterize the larval lethality phenotype and perform a comparison of the larval lethality phenotype to the glycerol hypersensitive phenotype at the level of GK phosphorylation activity, *dGyk*/*dGK*-RNA expression, and glycerol levels.

### Both dGyk and dGK are required for normal glycerol kinase activity levels

**Figure 1:**
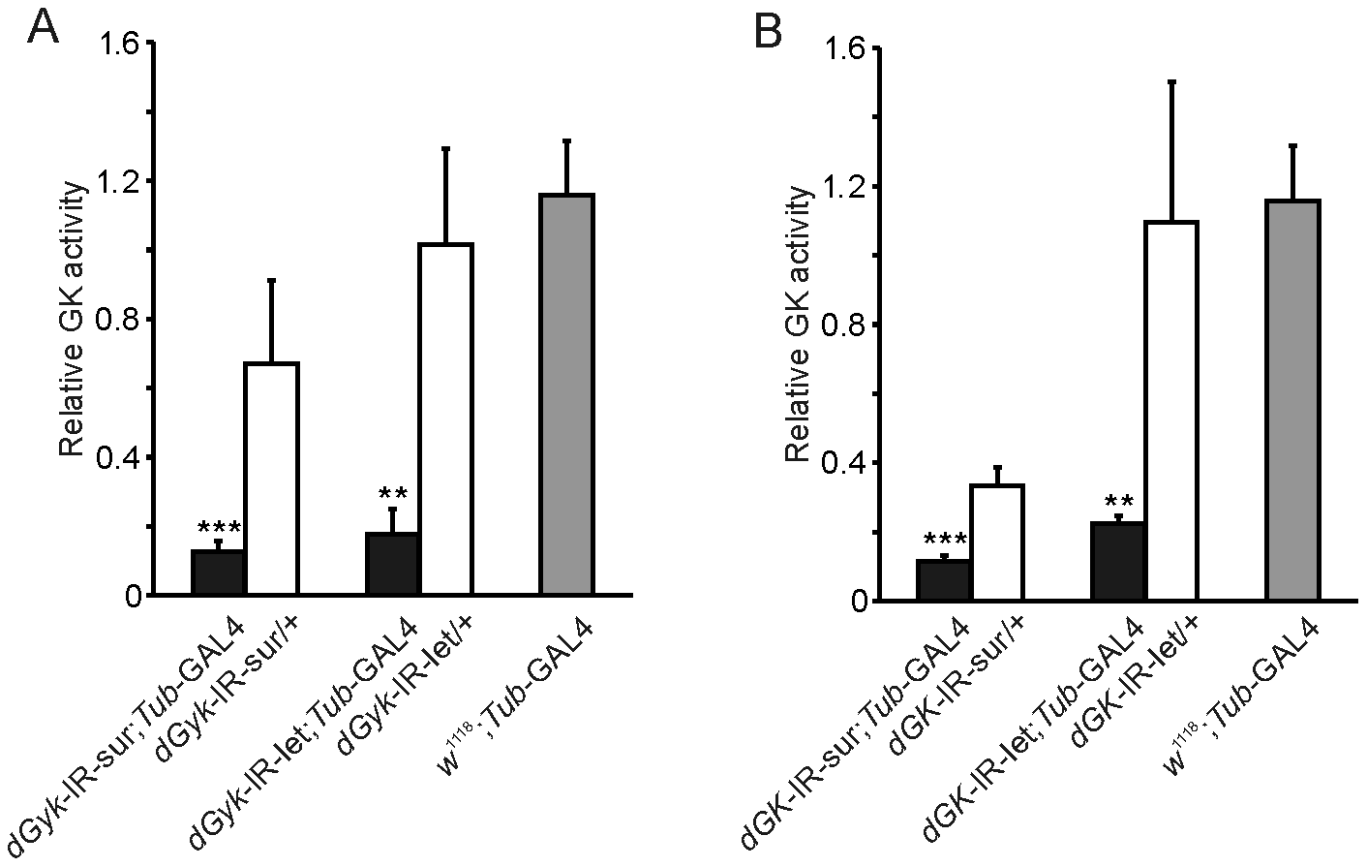
RNAi targeting of *dGyk* (A) or *dGK* (B) decreases glycerol kinase activity. (A) Glycerol kinase activity was reduced in both *dGyk*-IR-sur; *Tub*-GAL4 and *dGyk*-IR-let; *Tub*-GAL4 3^rd^ instar larvae. (B) Glycerol kinase activity was reduced in both *dGK*-IR-sur; *Tub*-GAL4 and *dGK*-IR-let; *Tub*-GAL4 3^rd^ instar larvae. Parental controls (*w^1118^*; *dGyk*-IR, *w^1118^*; *dGK*-IR and *w^1118^*; *Tub*-GAL4) were statistically similar (*w^1118^*; *Tub*-GAL4 shown). Abbreviations: “sur” and “let” refer to progeny that survive to adulthood or show lethality before eclosion, respectively. Error bars represent standard error between biological replicates. Statistical analysis using ANOVA was performed by comparison to parental controls. ** *P*<0.01, ***P<0.001.

Glycerol kinase (GK) phosphorylates glycerol to glycerol 3-phosphate. Therefore successful targeting of *dGyk* or *dGK* should result in decreased GK activity. Using radiolabelled ^14^C glycerol to assay for glycerol kinase (GK) phosphorylation activity, we found decreased but similar levels of GK activity for *dGyk*-IR-sur; *Tub*-GAL4, *dGyk*-IR-let/*Tub*-GAL4, *dGK*-IR-sur; *Tub*-GAL4, and *dGK*-IR-let/*Tub*-GAL4 3^rd^ instar RNAi progeny (Figure 1). This result indicates both dGyk and dGK are required for normal levels of GK glycerol phosphorylating activity.

### Alternative phenotypes have distinct *dGyk* and *dGK* expression levels

**Figure 2:**
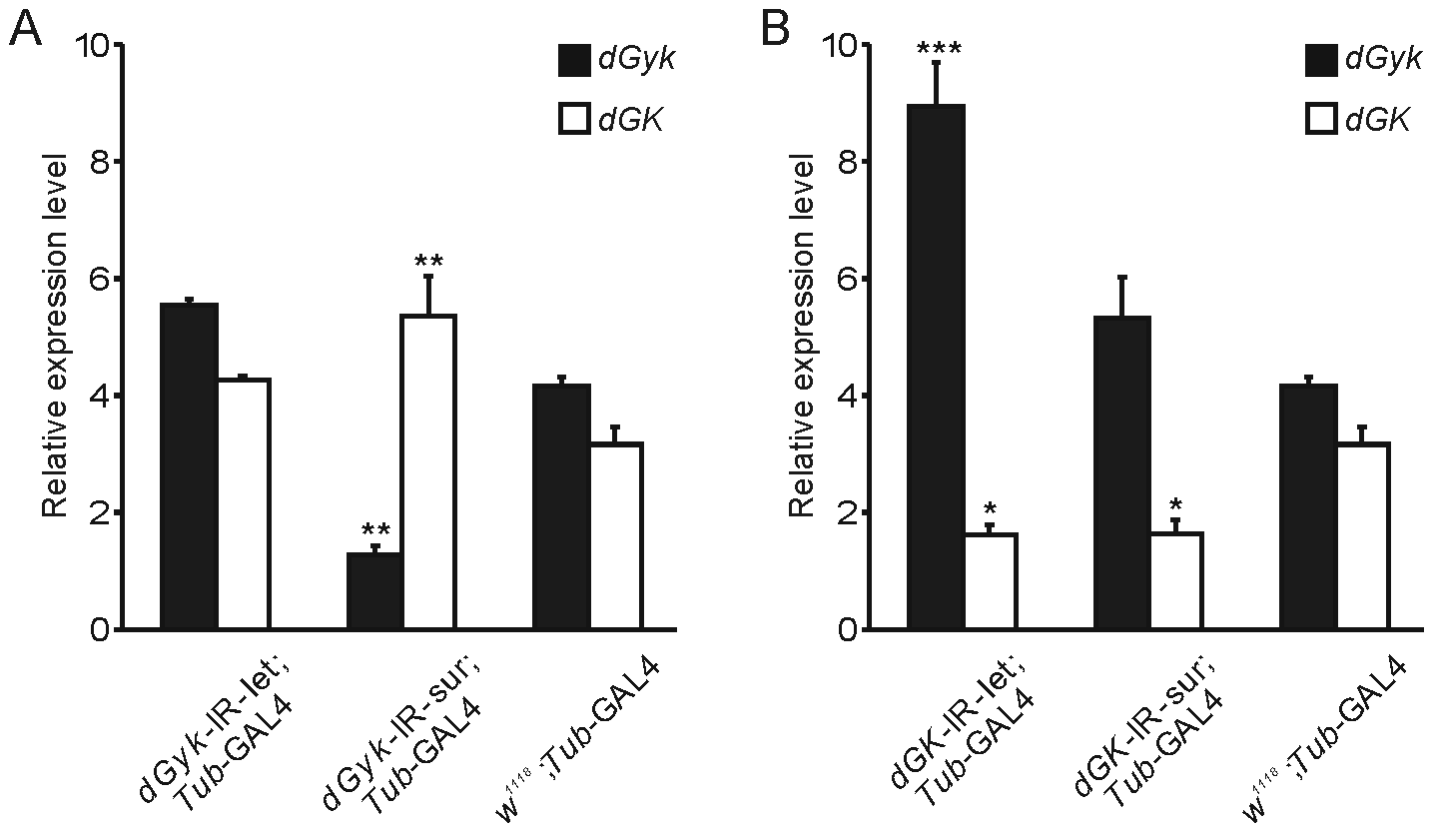
RNA quantification supports a compensatory mechanism between *dGyk* and *dGK*. RNA expression levels were determined by qRT-PCR for: (A) *dGyk*-IR-sur; *Tub*-GAL4 and *dGyk*-IR-let; *Tub*-GAL4 3^rd^ instar larvae, and (B) *dGK*-IR-sur; *Tub*-GAL4 and *dGK*-IR-let; *Tub*-GAL4 3^rd^ instar larvae. This analysis revealed *dGyk*-IR-sur; *Tub*-GAL4 and *dGK*-IR-sur; *Tub*-GAL4 to have decreased levels of *dGyk* and *dGK*, respectively. Interestingly, while *dGK*-IR-let/*Tub*-GAL4 showed reduced *dGK* expression, a significant increase in *dGyk* levels was also detected indicating the existence of a compensatory mechanism at the RNA level between *dGK* and *dGyk*. This is also supported by the *dGyk*-IR-sur; *Tub*-GAL4 result that shows increased *dGK* levels in addition to the expected decreased levels of *dGyk*. Unexpectedly, the expression levels of *dGyk* and *dGK* in the *dGyk*-IR-let/*Tub*-GAL4 progeny were relatively unchanged compared to controls (see discussion). RNA levels for parental construct fly lines (*w^1118^*; *dGyk*-IR, *w^1118^*; *dGK*-IR) were also determined but were not significantly different from the *w^1118^*; *Tub*-GAL4 control (Figure S1). Statistical analysis using ANOVA was performed by comparison to parental controls. *P<0.05, **P<0.01, ***P<0.001.

We used qRT-PCR to determine RNA expression levels of *dGyk* and *dGK* in RNAi progeny from *Tub*-GAL4 crosses (Figure 2). This revealed *dGyk*-IR-sur; *Tub*-GAL4 and *dGK*-IR-sur; *Tub*-GAL4 to have decreased levels of *dGyk* and *dGK*, respectively. Interestingly, while *dGK*-IR-let/*Tub*-GAL4 showed reduced *dGK* expression, a significant increase in *dGyk* levels was also detected indicating the existence of a compensatory mechanism at the RNA level between *dGK* and *dGyk*. This observation also is supported by the *dGyk*-IR-sur; *Tub*-GAL4 result that shows increased *dGK* levels in addition to the expected decreased levels of *dGyk*. Unexpectedly, the expression levels of *dGyk* and *dGK* in the *dGyk*-IR-let/*Tub*-GAL4 progeny were relatively unchanged as compared to controls. This intriguing result could be caused by inhibition of RNAi triggered by cell death in adjacent cells (see discussion). Relative RNA expression levels of *dGyk* and *dGK* were quantitated for parental fly lines used to generate RNAi knockdown flies (Figure S1).

### High glycerol levels correlate with glycerol hypersensitivity, whereas low glycerol levels correlate with larval lethality

**Figure 3:**
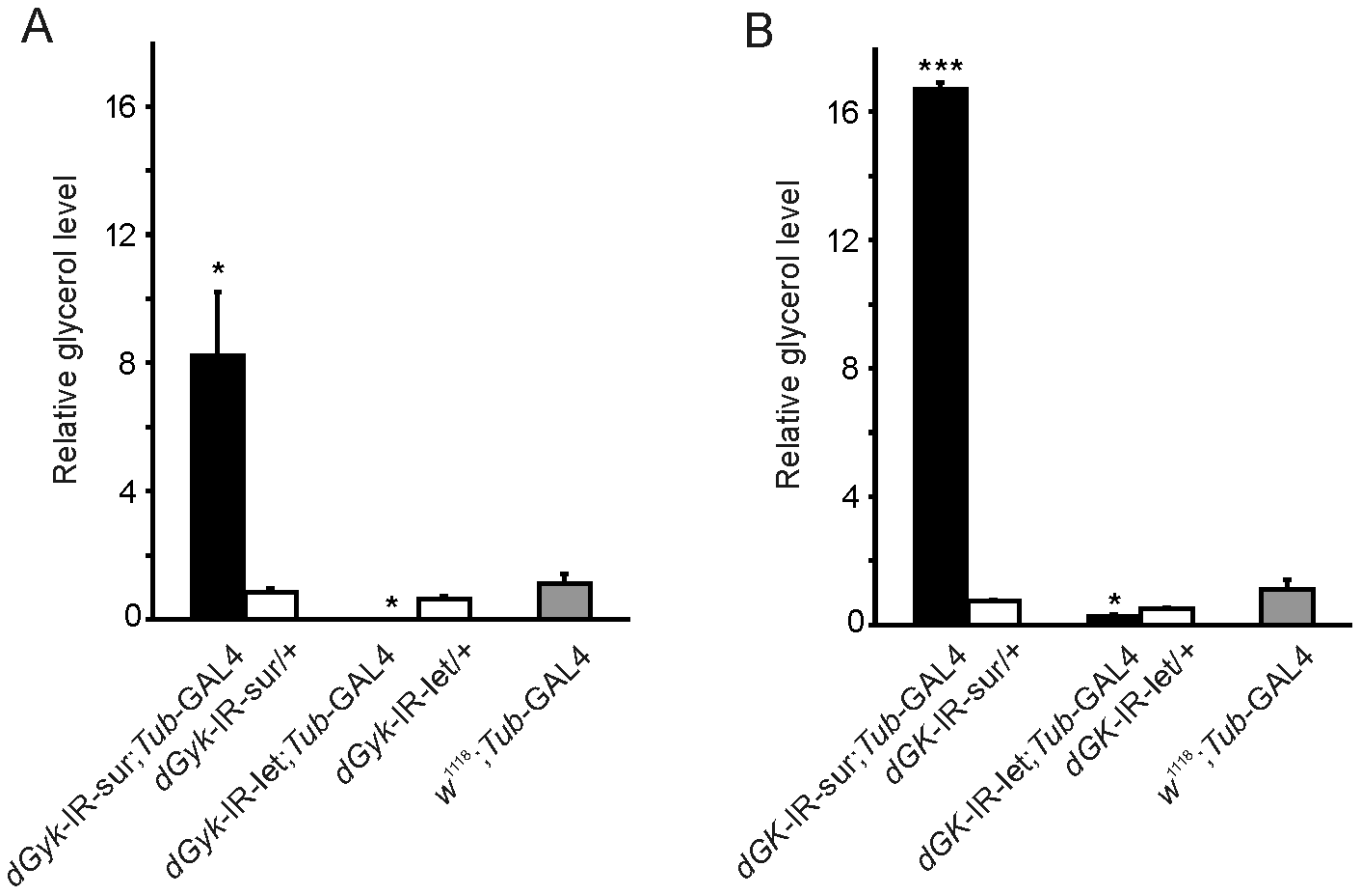
Distinct glycerol levels correlate with phenotype. Glycerol levels were determined for: (A) *dGyk*-IR-sur; *Tub*-GAL4 and *dGyk*-IR-let; *Tub*-GAL4 3^rd^ instar larvae, and (B) *dGK*-IR-sur; *Tub*-GAL4 and *dGK*-IR-let; *Tub*-GAL4 3^rd^ instar larvae. Elevated glycerol levels were found for “sur” offspring while decreased glycerol levels for “let” genotypes compared to parental control 3^rd^ instar larvae *w^1118^*; *dGyk*-IR, *w^1118^*; *dGK*-IR and *w^1118^*; *Tub*-GAL4 (*w^1118^*; *Tub*-GAL4 shown). Statistical analysis using ANOVA was performed by comparison to parental controls. *P<0.05, ***P<0.001.

Glycerol kinase phosphorylates glycerol to glycerol 3-phosphate in an ATP dependent reaction. Therefore, with decreased GK activity (as defined as glycerol phosphorylation) we would anticipate elevated glycerol levels. As expected, we found increased levels of glycerol in *dGyk*-IR-sur; *Tub*-GAL4 and *dGK*-IR-sur; *Tub*-GAL4 3^rd^ instar larvae (Figure 3). These larvae develop into glycerol hypersensitive adult flies. Intriguingly, *dGyk*-IR-let/*Tub*-GAL4 and *dGK*-IR-let/*Tub*-GAL4 had deceased levels of glycerol, suggesting the lack of glycerol might contribute to the lethality phenotype. Triglyceride levels of RNAi progeny were indistinguishable from those of controls (data not shown). Data for GK activity, RNA expression, glycerol levels are summarized in Table 1.

**Table 1:** Summary of RNAi data.

RNAi	GAL4	Lethality	Relative level compared to control			
line	driver	before	RNA level		GK activity	Glycerol
		eclosion?	*dGyk*	*dGK*		
*dGyk*-IR-sur	*Tubulin*	No	−−	+	−−−	++
*dGyk*-IR-let	*Tubulin*	Yes	normal	normal	−−	−−−
*dGK*-IR-sur	*Tubulin*	No	normal	−−	−−−	+++
*dGK*-IR-let	*Tubulin*	Yes	++	−−	−−	−−

Analysis was performed on RNAi; *Tubulin*-GAL4 3^rd^ instar larvae. +/− increased or decreased levels.

Additionally, we quantitated hemolymph trehalose levels (trehalose is the principal blood sugar in insects). In humans, expression of glycerol kinase is highest in the liver [17]. Therefore, we used the *c564*-GAL4 driver that has previously been shown to drive expression of GAL4 in the larval fat body [23], [24], a tissue that plays an important role in energy metabolism similar to that of mammalian liver [25]. Quantitation of trehalose revealed decreased levels in both *c564*-GAL4; *dGyk*-IR-let; and c564-GAL4; *dGK*-IR-let 3^rd^ instar larvae but unchanged levels in *c564*-GAL4; *dGyk*-IR-sur and *c564*-GAL4; *dGK*-IR-sur 3^rd^ instar larvae (Figure S2).

### Characterization of lethality and wing phenotypes

**Figure 4:**
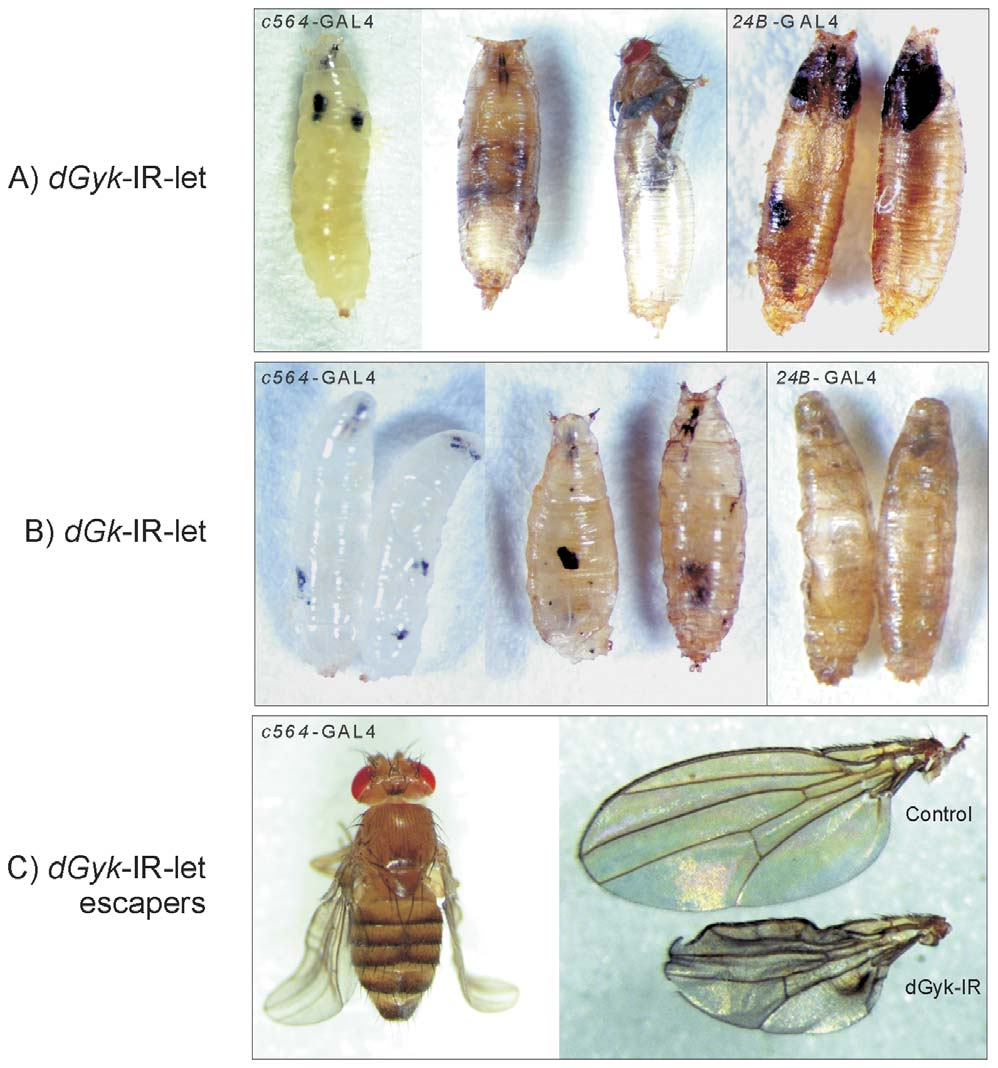
Developmental phenotypes displayed by RNAi targeting of *dGyk* or *dGK*. Progeny from (A) *dGyk*-IR-let and (B) *dGK*-IR-let flies result in larvae with melanotic masses and lethality at the larval or pupal stage of development for both *c564*-GAL4 or *24B*-GAL4 drivers. (C) *c564*-GAL4; *dGyk*-IR-let escaper flies had curled/crumpled wings with dark pigmented areas. Note: For *c564*-GAL4; *dGyk*-IR-let progeny, the majority die before eclosion (80%). For *c564*-GAL4; *dGK*-IR-let offspring, 100% lethality was observed before eclosion.

Using a variety of GAL4 drivers with different expression profiles, we performed phenotypic screening of all the *dGyk*-IR-sur, *dGK*-IR-sur, *dGyk*-IR-let, and *dGK*-IR-let fly lines. GAL4 drivers tested included *c564* (larval fat body), *24B* (embryonic mesoderm and muscle), *Elav* (nervous system), and *GMR* (eye). In addition to the larval lethality phenotype obtained in progeny from *dGyk*-IR-let and *dGK*-IR-let with the *Tub*-GAL4 driver, we found lethality at larval and pupal stages of development for RNAi progeny from *dGyk*-IR-let and *dGK*-IR-let fly lines using *c564*-GAL4 and *24B*-GAL4 driver crosses (Figure 4A and 4B). Progeny from the *dGyk*-IR-sur and *dGK*-IR-sur lines did not have any physical phenotype for any of the GAL4 drivers tested. Therefore only RNAi lines that resulted in lethal outcomes with the *Tub*-GAL driver also resulted in physical phenotypes with the *c564*-GAL4 and *24B*-GAL4 drivers. 3^rd^ instar larval progeny (*c564*-GAL4; *dGyk*-IR-let and *c564*-GAL4; *dGK*-IR-let) often exhibited melanotic masses before lethality at the pupal stage of development. The majority of *c564*-GAL4; *dGyk*-IR-let progeny die as pharate adults (80% lethality), with escapers exhibiting a curled or crumpled wing phenotype (Figure 4C). Lethality of *c564*-GAL4; *dGK*-IR-let progeny was 100% penetrant at the pupal stage. No external or behavioral phenotype was observed in RNAi offspring from *Elav*-GAL4 and *GMR*-GAL4 driver crosses.

### Lethality phenotype rescued by transgenic over-expression constructs

**Figure 5:**
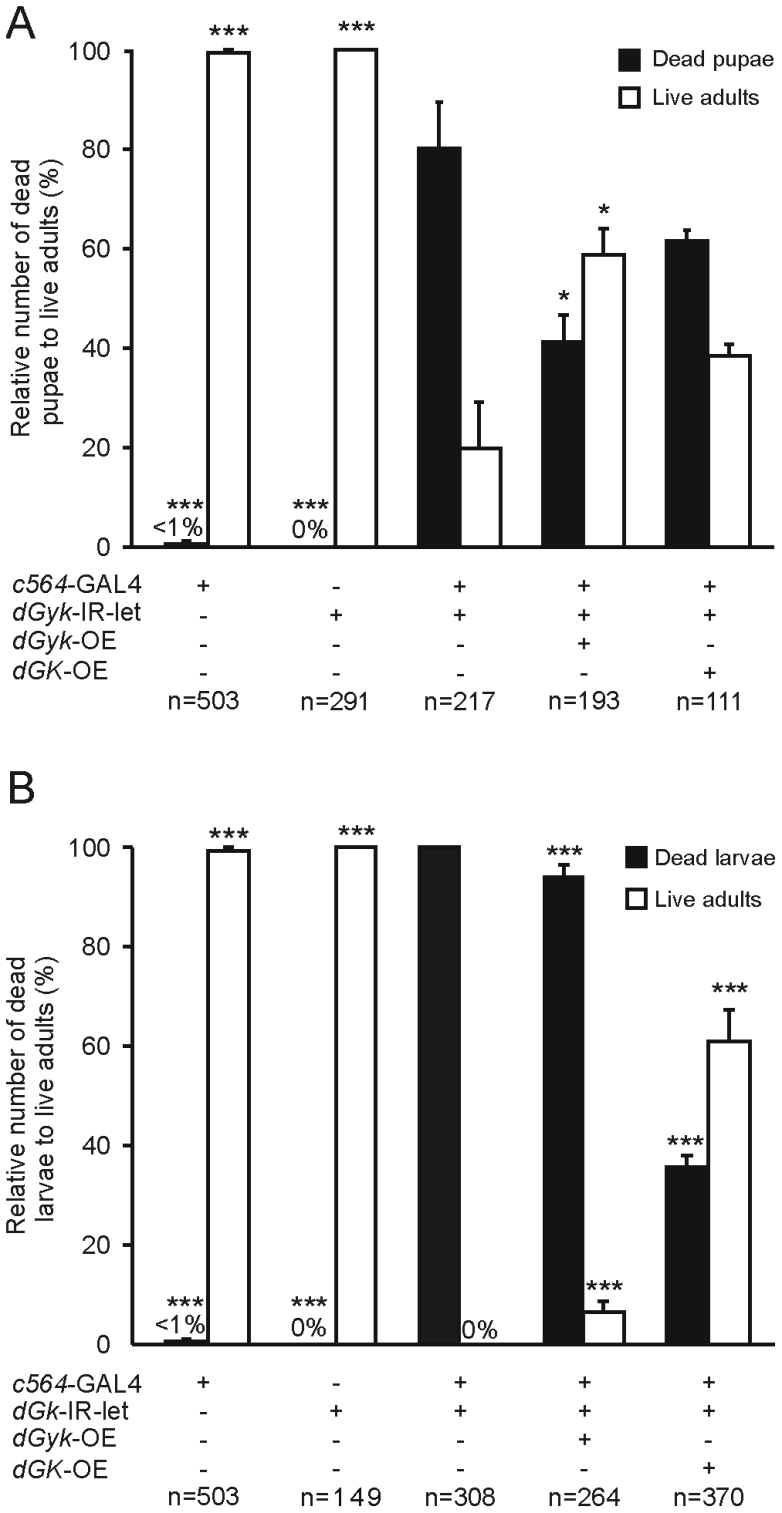
Rescue of lethality phenotype by transgenic over-expression of *dGyk* and *dGK*. (A) The *c564*-GAL4; *dGyk*-IR-let lethality phenotype results in ∼80% of progeny dying at the pupal stage of development. Rescue by *dGyk*-OE reduces lethality to 41% during pupal development. Attempted rescue by *dGK*-OE reduces lethality to 59% (value not significant). (B) The *c564*-GAL4; *dGK*-IR-let genotype results in 100% lethality before eclosion with only 15% of progeny developing into roaming larvae. Rescue by *dGK*-OE reduces lethality to 36%. Rescue by *dGyk*-OE reduces lethality to 94%. Rescue experiments were performed in triplicate and error bars represent SEM. *P* values were calculated for living adult progeny for each genotype using *c564*-GAL4; RNAi as control. *P<0.05, **P<0.01, ***P<0.001.

In order to provide evidence supporting lethality during larval/pupal development of *c564*-GAL4; *dGyk*-IR-let and *c564*-GAL4; *dGK*-IR-let progeny was due to altered *dGyk* and *dGK* expression levels, we performed rescue of phenotype experiments using transgenic over-expression constructs *dGyk*-OE and *dGK*-OE (Figure 5). Previous analysis of *dGyk*-OE and *dGK*-OE transgenic flies confirmed over-expression at the RNA level of *dGyk* and *dGK* respectively [19]. Penetrance of pupal lethality for *c564*-GAL4; *dGyk*-IR-let was significantly reduced from 80% to 41% by *dGyk*-OE (Figure 5A). In the case of *c564*-GAL4; *dGK*-IR-let progeny that exhibit 100% lethality during larval development, rescue by *dGK*-OE successfully reduced lethality to 36% (Figure 5B).

### Severity of wing phenotype enhanced by mutation of *dGpdh*

**Figure 6:**
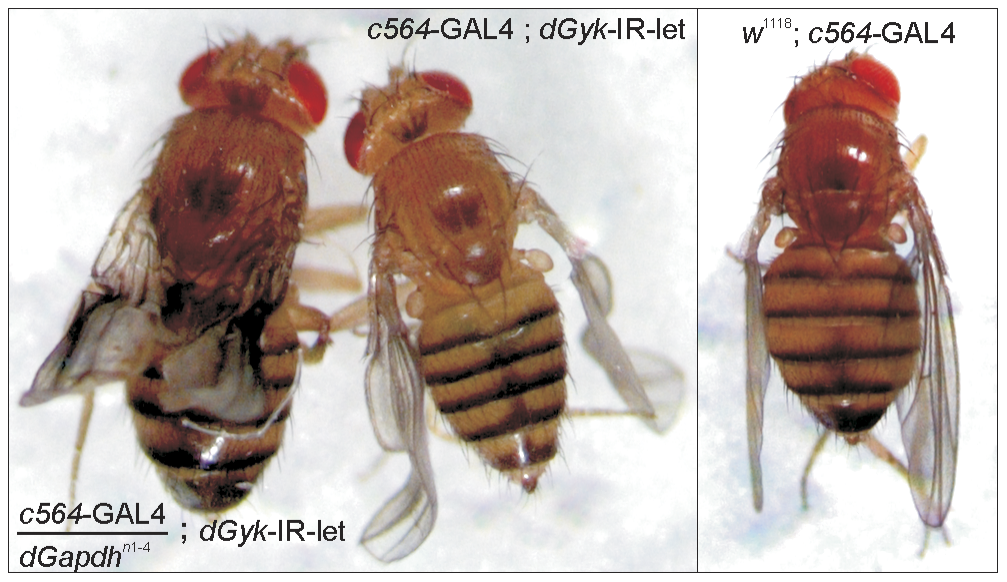
Wing phenotype is enhanced by a loss of function allele of *dGpdh*. Wings of *c564*-GAL4/*dGpdh^n1-4^*; *dGyk*-IR-let flies were found to have a more severe wing phenotype as compared to *c564*-GAL4; *dGyk*-IR-let flies. Control *w^1118^*; *c564*-GAL4 flies had normal wings. This result indicates the *c564*-GAL4; *dGyk*-IR-let wing phenotype is likely due to disrupted glycerol metabolism and not an alternative function of glycerol kinase.

To determine whether the wing phenotype displayed by *c564*-GAL4; *dGyk*-IR-let escaper flies was due to disrupted glycerol metabolism or an alternative function of the glycerol kinase protein, we crossed *c564*-GAL4; *dGyk*-IR-let flies with a loss of function allele for the glycerol 3-phosphate dehydrogenase gene (*dGpdh^n1-4^*). As both dGyk and dGpdh play important enzymatic roles in glycerol metabolism, we would predict that the *dGpdh^n1-4^* mutation would enhance the *c564*-GAL4; *dGyk*-IR-let wing phenotype if the phenotype was caused by disrupted glycerol metabolism. Crosses were performed between *c564*-GAL4; *dGyk*-IR-let and *dGpdh^n1-4^* flies and progeny examined for the crumpled wing phenotype. Wings of *c564*-GAL4/*dGpdh^n1-4^*; *dGyk*-IR-let flies were found to have a more severe wing phenotype compared to *c564*-GAL4; *dGyk*-IR-let flies (Figure 6). Therefore the *c564*-GAL4; *dGyk*-IR-let wing phenotype is likely due to disrupted glycerol metabolism.

## Discussion

In humans, GKD patients show a range of phenotypic severity with no correlation with GK glycerol phosphorylation activity. This has led to the hypothesis of an important role for modifier loci and/or alternative protein functions of glycerol kinase in determining phenotype severity. Remarkably, our *Drosophila* model for GKD also results in a range of phenotype severity that includes glycerol hypersensitive adults and lethality during larval development. We had previously shown GFP levels (the RNAi construct co-expresses GFP with the inverted repeat sequence of the target gene) to be elevated in larvae that die before eclosion compared to the glycerol hypersensitive adults [19]. Dosage sensitivity is a feature of a number of metabolic related genes, e.g., expression of the mouse *OB1* gene (homolog of the human gene encoding leptin) in relation to obesity [26]. Therefore we expected glycerol kinase activity and expression levels of *dGyk* or *dGK* to be lower for the lethality phenotype as compared to the glycerol hypersensitive phenotype. However, here we show that the underlying molecular basis has a greater level of complexity, a characteristic shared with GKD patients.

At the amino acid level, dGyk and dGK are 46% identical (67% similar if including conservative substitutions) and share the “FGGY” domain responsible for glycerol phosphorylation [27], [28]. The overlapping function between dGyk and dGK is supported by the similar RNAi phenotypes of glycerol hypersensitivity and larval lethality. Furthermore, the phenotypic rescue experiment showed that over-expression of *dGyk* can partially rescue lethality of *c564*-GAL4; *dGK*-IR-let. Future studies correlating phenotype to dosage levels between dGyk and dGK could provide an interesting insight into the individual functions of dGyk and dGK. The presence of other distinct protein domains within the dGyk and dGK amino acid sequence, e.g., domains for protein interaction and mitochondrial apoptosis [29], suggests that dGyk and dGK are likely to possess additional and non-overlapping functions. However the significance and function of these protein domains is currently unknown.

For the *dGyk* and *dGK* glycerol hypersensitive and larval lethality phenotypes, glycerol kinase activities showed a trend toward reduction. However, distinct *dGyk*- and *dGK*-RNA expression profiles were found between the glycerol hypersensitive and larval lethality phenotypes. One notable feature was a compensatory mechanism between *dGyk* and *dGK*. We observed that the *dGyk*-IR-sur; *Tub*-GAL4 flies had reduced levels of *dGyk* and elevated levels of *dGK*, whereas *dGK*-IR-let; *Tub*-GAL4 showed reduced levels of *dGK* and elevated levels of *dGyk*. This compensatory mechanism was at the level of RNA expression and did not restore GK activity to normal levels. These results indicate that both dGyk and dGK are required for normal levels of GK activity. In bacteria, the glycerol kinase protein can exist as a dimer or tetramer with each state affecting the protein conformation and glycerol kinase activity [30]. However, it is unknown if dimerization can occur between dGyk and dGK and whether this can affect glycerol kinase activity in *Drosophila*.

All the *Tub*-GAL4; *dGyk*-IR and *Tub*-GAL4; *dGK*-IR flies had decreased levels of GK phosphorylation activity. However, RNA expression analysis of *dGyk*-IR-let; *Tub*-GAL4 3rd instar larvae unexpectedly revealed levels of *dGyk* that were not statistically different as compared to controls. We hypothesize that this is due to inhibition of the RNAi mechanism. Recent studies have shown that RNAi constructs that trigger apoptotic cell death can result in RNAi inhibition in adjacent cells [31], [32]. Therefore, in the case of *dGyk*-IR-let; *Tub*-GAL4 flies, cell specific RNAi inhibition could mask RNAi knockdown of *dGyk*-RNA levels in other cells. However without experimental confirmation this remains speculation.

*In silico* analysis of the *dGyk*-IR and *dGK*-IR construct sequences did not identify any potential off-targets in the *Drosophila* genome (see methods for analysis details). Additionally, *dGyk*-IR does not target the *dGK* transcript and the *dGK*-IR does not target the *dGyk* transcript. However, without a dGyk-specific antibody to perform immunohistochemistry, we have been unable to confirm dGyk knockdown at the protein level in the *dGyk*-IR-let; *Tub*-GAL4 flies (although GK phosphorylation activity is decreased). The fact that *dGyk*-IR-let; *Tub*-GAL4 flies had reduced GK activity and a phenotype resembling that of the *dGK*-IR-let; *Tub*-GAL4 flies suggests that total dGyk protein levels are reduced.

The metabolic role of glycerol kinase is to phosphorylate glycerol to glycerol 3-phosphate in an ATP dependent reaction. Therefore, with decreased GK activity we would anticipate elevated glycerol levels. As expected, elevated glycerol levels were found in *dGyk*-IR-sur; *Tub*-GAL4 and *dGK*-IR-sur; *Tub*-GAL4 flies. Interestingly, both *dGyk*-IR-let; *Tub*-GAL4 and *dGK*-IR-let; *Tub*-GAL4 flies had glycerol levels that were lower than control levels. Further evidence for altered metabolite levels was obtained by quantitation hemolymph trehalose. Decreased trehalose levels were found in both *c564*-GAL4; *dGyk*-IR-let; and c564-GAL4; *dGK*-IR-let 3^rd^ instar larvae whereas trehalose levels were unchanged in *c564*-GAL4; *dGyk*-IR-sur and *c564*-GAL4; *dGK*-IR-sur 3^rd^ instar larvae. We hypothesize that reduced glycerol and trehalose is part of the pathogenic mechanism in which disrupted metabolism contributes to larval lethality. Future comprehensive metabolic profiling could reveal clues to the underlying pathogenic mechanism.

As all the knockdown flies had normal triglyceride levels, we also predict that glycerol utilization through an alternative metabolic pathway could contribute toward the deleterious outcome of larval lethality. For example, future studies are required to determine whether reduced glycerol kinase activity alters di-acylglycerol (DAG) levels. DAG can bind a number of signaling proteins that affect a variety of cellular processes such as cytoskeletal reorganization, membrane trafficking, exocytosis, immune synapse formation, synaptic transmission and phagocytosis [33], [34]. Levels of DAG can be controlled by di-acylglycerol kinases (DGK) by conversion of DAG to phosphatidic acid (PA) whereby PA itself can also affect a number of cellular processes [35]. Therefore, altered levels of DAG and PA could provide a link to signaling pathways and the pathogenic mechanism underlying the *Drosophila* GKD phenotypes.

The larval lethality and crumpled wing phenotypes (Figure 4) also suggest a link to signaling pathways. For example, the appearance of melanotic masses in larvae before death is consistent with the activation of cell death signaling pathways [36]. The identification of a crumpled wing phenotype in escaper flies for *c564*-GAL4; *dGyk*-IR-let flies could be as a result of altered cell signaling pathways. Wing phenotypes in *Drosophila* can arise when cell signaling pathways such as Notch signaling pathway are affected [37], [38]. Identification of modifiers of the wing phenotype has the potential to identify a link between glycerol kinase activity and signaling pathways.

In humans, the study of GKD patients clearly demonstrates an important role for glycerol kinase in development [1]. The identification of the crumpled wing phenotype exhibited by *c564*-GAL4; *dGyk*-IR-let escaper flies in addition to larval lethality shows glycerol kinase also plays an important role in *Drosophila* development. To determine whether the crumpled wing phenotype was due to disrupted glycerol metabolism or due to loss of an alternative function of dGyk, we used another *Drosophila* mutant with disrupted glycerol metabolism. Using a loss of function allele in the glycerol 3-phosphate dehydrogenase 1 gene (*dGpdh^n1-4^*) we were able to show that *c564*-GAL4/*dGpdh^n1-4^*; *dGyk*-IR-let flies had a more severe wing phenotype than *c564*-GAL4; *dGyk*-IR-let flies. Therefore we conclude that the wing phenotype is due to disruption of glycerol metabolism.

Both glycerol kinase and glycerol 3-phosphate dehydrogenase control levels of glycerol 3-phosphate, a precursor for phospholipid biosynthesis. Interestingly, mutations in glycerol 3-phosphate dehydrogenase (*GPDH1*) result in transient infantile hypertriglyceridemia, fatty liver, and hepatic fibrosis [39]. Further study of the flies with disruption of both *dGyk* and *dGpdh1* expression levels is required to determine how glycerol metabolism is affected and whether this could provide clues to the pathogenic mechanism underlying this crumpled wing phenotype.

Taken together, these data demonstrate that disruption of glycerol metabolism by RNAi targeting of either glycerol kinase gene, *dGyk* or *dGK*, results in a range of phenotypic severity. Our initial characterization of the glycerol hypersensitivity and larval lethality phenotypes reveals a level of complexity in the underlying pathogenic mechanism similar to that observed in human GKD patients. The identification of a crumpled wing phenotype suggests cell signaling could be affected. Therefore, this *Drosophila* model for GKD is worthy of further investigation and could provide novel insights into the underlying pathogenetic mechanism observed in human GKD patients.

## Materials and Methods

### Constructs and *Drosophila* stocks

Using the *UAS*/GAL4 system [40]–[42], RNAi and over-expression constructs for *dGyk* and *dGK* were created as previously described [19]. Briefly, cDNA fragments were PCR amplified from Berkeley Drosophila Genome Project cDNA clones GH12641 and GH18680 that contain complete coding regions for *dGyk* and *dGK* respectively. For RNAi constructs, PCR amplified cDNAs were initially subcloned into the *pHIBS* vector before further subcloning as an inverted repeat (IR) into the *pUDsGFP* vector [43]. The *pUDsGFP* construct co-expresses GFP with the inverted repeat, allowing easy recognition of GFP-positive larvae that possess both the RNAi construct and the GAL4 driver. Primer pairs for PCR amplification were as follows: dGyk-IR-for d5′- AGTTGGATCCGAAATAATCACGATTGGAA -3′ and dGyk-IR-rev d5′- AGTTGGTACCTAGTAATCCGTGCGTTGAG-3′; dGK-IR-for d5′- AGTTGGATCCCTGCTCAAGACGTTCGGTA -3′ and dGK-IR-rev d5′- AGTTGGTACCTCGAACTGGCAGAGATTGA -3′. Evaluation of the inverse repeat sequences using the online web application E-RNAi version 3.2: http://www.dkfz.de/signaling/e-rnai3// [44] did not identify any off-targets in the *Drosophila* genome. Additionally, *dGyk*-dsRNA does not target the *dGK* transcript and the *dGK*-dsRNA does not target the *dGyk* transcript.

For over-expression constructs, the complete coding regions for *dGyk* and *dGK* were PCR amplified and subcloned into the *pEx-UAS* vector [45]. Primers for PCR amplifying the complete coding regions for *dGyk* and *dGK* were as follows: dGyk-for d5′ATTGCGGCCGCAAAAAAAATGGATTCTCCC3′ and dGyk-rev d5′ATTTCTAGATGATCACGCTCCGTCAAAGGC3′; dGK-for d5′ATTGCGGCCGCAAGCAGCATGACCGAGGGC3′ and dGK-rev d5′AGCTCTAGATATTTACTGGCCACTCGCAGC3′. Microinjection of DNA constructs, identification of transformants and balancing were performed by BestGene Inc (Chino Hills, CA).

As described previously [19], analysis of *dGyk*-IR x *Tub*-GAL4 crosses revealed 3 lines that resulted in viable adult flies and 6 lines that resulted in progeny that died during larval development. For *dGK*-IR x *Tub*-GAL4 crosses, 7 lines resulted in viable adults flies and 2 lines resulted in progeny that died during larval development. Neither “let” nor “sur” transgenes were homozygous lethal. For all subsequent experiments, 2 fly lines for each RNAi phenotype were chosen for analysis (results are shown for single fly lines).

All GAL4 driver fly stocks were obtained from the BDSC: *P{TubP-GAL4}* [46]; *P{GawB}c564* [23]; *P{GawB}how[24B]* [40]; *P{GawB}Elav[C155]* [47]; *P{GMR-GAL4}* [48]. The glycerol phosphate dehydrogenase loss of function mutant was also obtained from BDSC: al^1^ *Gpdh^n1-4^*/SM1 [49]. For all fly crosses, progeny were genotyped based on presence or absence of balancer chromosome markers.

### Glycerol kinase activity assay

Glycerol kinase activity was determined using a radiolabelled assay as previously reported [50]. Briefly, protein was extracted in homogenization buffer (1% KCl; 1 mM EDTA+ Complete protease inhibitor (Roche, Indianapolis, IN)) from two groups of three 3^rd^ instar larvae and assayed in duplicate using 4 µg of total cellular protein for 20 min using assay conditions and reaction mix previously determined to be optimal for 3^rd^ instar larvae protein extracts (data not shown). Incorporation of ^14^C-glycerol (GE Healthcare, Piscataway, NJ) into glycerol 3-phosphate was measured using a scintillation counter and GK activity of test samples calculated by comparison to a standard curve.

### RNA preparation and quantitative real-time PCR

RNA was extracted from ten 3^rd^ instar larvae using the RNAeasy® mini kit (Invitrogen, Carlsbad, CA) according to manufacturer's instructions. Total RNA (1 µg) was used for first-strand cDNA synthesis using the SuperScript® III reverse transcriptase and random primers (Invitrogen). Quantitative real-time PCR (qRT-PCR) was performed using PerfecCTa™ SYBR® Green FastMix™ ROX (Quanta Biosciences, Gaithersburg, MD) on a StepOne™ real time PCR machine (Applied Biosystems, Foster City, CA). Fold differences for each of the genes tested were calculated using the 2[Delta][Delta]CT method [51]. All reactions were performed in triplicate. Expression levels of *dGyk* and *dGK* were normalized to *RpII*. Primers were designed using Primer3 software [52] and synthesized by Integrated DNA Technologies (San Diego, CA). Primer sequences were as follows: *dGyk* d5′TAGGCATAACATCGGTTCTGG3′ and d5′GCCTTCCGTCCTAGTTGGTAG-3′; *dGK* d5′AGACGACAATCGTCTGGGATG3′ and d5′CACGATCTGCTCCACTGTAG3′; *RpII* d5′AAGGCTATGGTGGTGTCTGG3′ and d5′GCTTACCCTCCACGTTCTGT3′.

### Glycerol and triglyceride assay

For glycerol and triglyceride measurements, batches of three 3^rd^ instar larvae were homogenized in 250 µl homogenization buffer (10 mM Tris-HCl pH 7.4, 10 mM NaCl, 1 mM EDTA, 0.5% Triton X-100) including Complete protease inhibitor (Roche). Next, 14 µl of 20% triton X-100 was added to 186 µl of the sample. After heating at 70°C (5 mins) to inactivate endogenous enzymes, samples were centrifuged at 13000 rpm (5 mins) and the supernatant transferred to a new tube (after homogenizing the white lipid ring with the tip of the pipette). Glycerol levels were measured using Free Glycerol Reagent (Sigma-Aldrich). Values were normalized against protein concentration using the Micro BCA™ Protein Assay Kit (Thermo Scientific, Rockford, IL) and experiments were performed in triplicate for each genotype.

### Statistical analysis

One way ANOVA with post-hoc pair wise multiple comparison procedures (Tukey Test) were applied to qRT-PCR and biochemical data where stated. Error bars represent SEM.

## Supporting Information

Figure S1 **Control RNA expression data for** **Figure 2** **.** Relative RNA expression levels of *dGyk* and *dGK* were quantitated for parental fly lines used to generate RNAi knockdown flies (A and B). For each group, values were not found to be statistically different. Statistical analysis using ANOVA was performed by comparison to GAL4 fly line.
(TIF)

Figure S2 **Hemolymph trehalose measurements.** **Relative** hemolymph trehalose levels in 3^rd^ instar larvae were determined for the following genotypes: *c564*-GAL4; *dGyk*-IR-sur, *c564*-GAL4; *dGyk*-IR-let, *c564*-GAL4; *dGK*-IR-sur, and *c564*-GAL4; *dGK*-IR-let. The control genotype was *w^1118^*; *c564*-GAL4. Both *c564*-GAL4; *dGyk*-IR-let and *c564*-GAL4; *dGK*-IR-let had decreased trehalose levels whereas trehalose levels were unchanged in *c564*-GAL4; *dGyk*-IR-sur and *c564*-GAL4; *dGK*-IR-sur 3^rd^ instar larvae. Statistical analysis using ANOVA was performed by comparison to the control *P<0.05, **P<0.01.
(TIF)

Methods S1 **Trehalose assay.**
(DOC)

Table S1 **Initial phenotypic characterization of RNAi fly lines using a Tub-GAL4 driver for ubiquitous expression.**
(DOCX)
