# Supplementary figures and images for: Disruption of Glycerol Metabolism by RNAi Targeting of Genes Encoding Glycerol Kinase Results in a Range of Phenotype Severity in Drosophila
Source: PLoS One. 2013 Sep 6;8(9):e71664. doi: 10.1371/journal.pone.0071664 (PMC3765373; doi:10.1371/journal.pone.0071664)

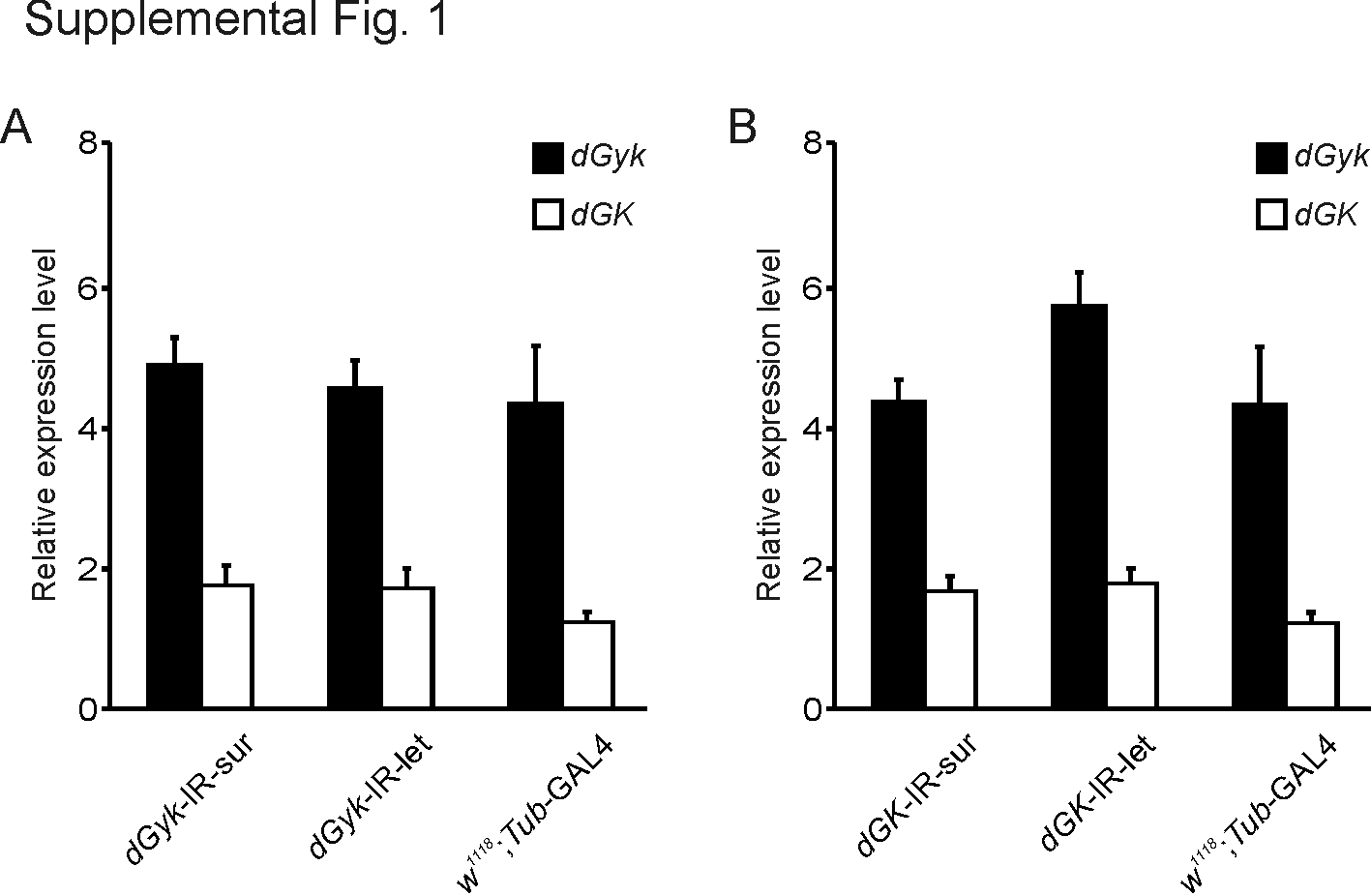

Supplement: Figure S1 — Control RNA expression data for Figure 2 . Relative RNA expression levels of dGyk and dGK were quantitated for parental fly lines used to generate RNAi knockdown flies (A and B). For each group, values were not found to be statistically different. Statistical analysis using ANOVA was performed by comparison to GAL4 fly line. (TIF) [file pone.0071664.s001.tif]

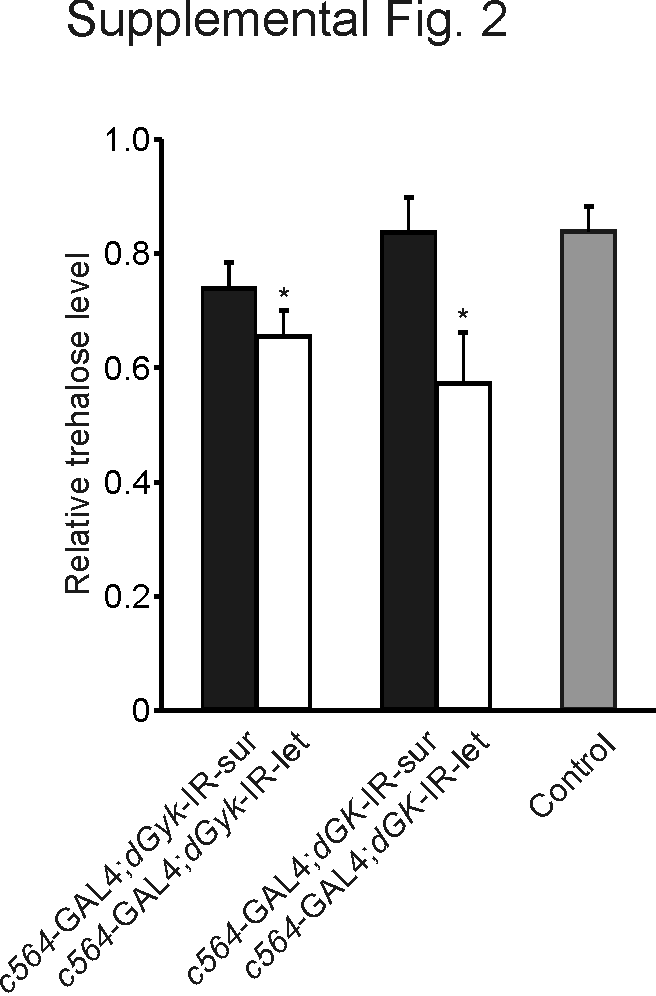

Supplement: Figure S2 — Hemolymph trehalose measurements. Relative hemolymph trehalose levels in 3rd instar larvae were determined for the following genotypes: c564-GAL4; dGyk-IR-sur, c564-GAL4; dGyk-IR-let, c564-GAL4; dGK-IR-sur, and c564-GAL4; dGK-IR-let. The control genotype was w1118; c564-GAL4. Both c564-GAL4; dGyk-IR-let and c564-GAL4; dGK-IR-let had decreased trehalose levels whereas trehalose levels were unchanged in c564-GAL4; dGyk-IR-sur and c564-GAL4; dGK-IR-sur 3rd instar larvae. Statistical analysis using ANOVA was performed by comparison to the control *P<0.05, **P<0.01. (TIF) [file pone.0071664.s002.tif]
