# Supplementary material for: Disruption of Glycerol Metabolism by RNAi Targeting of Genes Encoding Glycerol Kinase Results in a Range of Phenotype Severity in Drosophila
Source: PLoS One. 2013 Sep 6;8(9):e71664. doi: 10.1371/journal.pone.0071664 (PMC3765373; doi:10.1371/journal.pone.0071664)
Supplement: Methods S1 — Trehalose assay. (DOC) [file pone.0071664.s003.doc]

**Supplemental Methods**

**Trehalose assay**

Hemolymph was extracted from a group of five third instar larvae and 1 μl was put into 9 μl of buffer (5 mM Tris-HCl, pH 6.6, 137 mM NaCl, 2.7 nM KCl), heated at 70°C for 5 min before adding 1μl of trehalase (Sigma) and incubating at 37°C for 12 h. A commercial glucose (HK) assay kit (Sigma) was used to determine the trehalose level.

All assays were done at least in triplicate.
