# Supplementary material for: Disruption of Glycerol Metabolism by RNAi Targeting of Genes Encoding Glycerol Kinase Results in a Range of Phenotype Severity in Drosophila
Source: PLoS One. 2013 Sep 6;8(9):e71664. doi: 10.1371/journal.pone.0071664 (PMC3765373; doi:10.1371/journal.pone.0071664)
Supplement: Table S1 — Initial phenotypic characterization of RNAi fly lines using a Tub-GAL4 driver for ubiquitous expression. (DOCX) [file pone.0071664.s004.docx]

**Table S1.** Initial phenotypic characterization of RNAi fly lines using a *Tub*-GAL4 driver for ubiquitous expression. GAL4 progeny from both *dGyk*-IR and *dGK*-IR lines (IR: inverse repeat) survived to adulthood as glycerol hypersensitive adults (“sur”) or exhibited larval lethality (“let”).

| Fly line | GAL4 driver | Number of fly lines | Phenotype |
| --- | --- | --- | --- |
| *dGyk*-IR-sur | *Tubulin* | 3/9 | Glycerol hypersensitive |
| *dGyk*-IR-let | *Tubulin* | 6/9 | Larval lethality |
| *dGK*-IR-sur | *Tubulin* | 8/10 | Glycerol hypersensitive |
| *dGK*-IR-let | *Tubulin* | 2/10 | Larval lethality |
